# Correlation Between the First Trimester Two-Hour Postprandial Blood Glucose Greater Than 110 mg/dL for the Prediction of Gestational Diabetes Mellitus

**DOI:** 10.7759/cureus.66652

**Published:** 2024-08-11

**Authors:** Pikee Saxena, Akshma Yadav, Meenakshi Singh, Anjalakshi C., Rajeev Chawla, Hema Divakar, Veeraswamy Seshiah

**Affiliations:** 1 Obstetrics and Gynaecology, Lady Hardinge Medical College, New Delhi, IND; 2 Obstetrics and Gynaecology, Madras Medical College, Madras, IND; 3 Diabetology, North Delhi Diabetes Centre, Delhi, IND; 4 Obstetrics and Gynaecology, Divakar’s Specialty Hospital, Bengaluru, IND; 5 Diabetes and Endocrinology, The Tamil Nadu Dr. M.G.R. Medical University, Chennai, IND

**Keywords:** post-prandial blood glucose, first trimester, hba1c, gestational diabetes mellitus, prediction

## Abstract

Aim: To determine the correlation between first-trimester two-hour postprandial blood glucose (PPBG) > 110 mg/dL for predicting gestational diabetes mellitus (GDM).

Methods: This prospective cohort study enrolled 200 women between 8 and 10 weeks of gestation from February 2022 to February 2024. All recruited pregnant women underwent testing for two-hour PPBS at 8-10 weeks and were followed up till delivery. GDM screening was done during 14-16, 24-28, and 32-34 weeks of gestation.

Results: Amongst women having PPBS > 110 mg/dL, 95.9% developed GDM, while in the group with PPBS < 110 mg/dL, only 4% developed GDM. In the PPBS > 110 mg/dL group, a significantly higher number of women were in the older age group (p < 0.049), had a higher BMI (p < 0.001), a family history of diabetes (p < 0.001), and previous history of abortion (p < 0.001). Women with PPBS > 110 mg/dL had significantly higher rates of cesarean section (p < 0.01), preterm delivery (p < 0.001), and macrosomia (p < 0.001). A positive correlation (r = 0.677; p < 0.001) was observed between first trimester two-hour PPBS and cord blood glucose levels. Similarly, a positive correlation (r = 0.465; p < 0.001) was present between insulin levels measured during the first trimester with cord blood insulin. The area under the curve (AUC) for PPBS was 0.969 (p < 0.001) with 95% CI: 0.933-0.988. PPBS > 110 mg/dL has a sensitivity of 95.9%, specificity of 95.6%, positive predictive value (PPV) of 95.9%, negative predictive value (NPV) of 95.7%, and diagnostic accuracy of 95.77% to predict GDM.

Conclusion: PPBS > 110 mg/dL at two hours exhibits high levels of diagnostic accuracy for the prediction of GDM and is associated with adverse fetomaternal outcomes. PPBG is a superior, physiologic, and low-cost option compared to HbA1c for early prediction of GDM and can also be performed as a simple point-of-care test with a glucometer at home or in the periphery by healthcare workers (HCW) and in wellness centers.

## Introduction

As diabetes has become a pandemic of unprecedented proportions, it is now time to shift focus to its primordial prevention. For diabetes prevention, the first and foremost step is to have a reliable test for detecting and predicting gestational diabetes mellitus (GDM) so that appropriate, timely intervention may be initiated.

Hernandez et al. [1] studied the pattern of glycaemia in normal pregnancy by pooling analysis of 12 studies involving 45 years of data. They observed that the glycaemic targets in the management of hyperglycemia in pregnancy need to be lower than the currently used ones. The authors proposed lowering the fasting level to 71±8 mg/dL and two-hour postprandial < 110 mg/dL. 

A recent National Institutes of Health (NIH) study observed that HbA1C > 5.3% during 8-12 weeks of gestation predicts GDM [2,3]. Hemoglobin A1C (HbA1C) measured within the first trimester is helpful for early prediction of GDM. Each 0.1% (1 mmol/mol) rise in HbA1c at 8-13 weeks is associated with an adjusted 22% increased GDM risk [2].

However, HbA1c is not a feasible or practical test in resource-constrained settings. Recent publications state that, if postprandial blood glucose (PPBG) is > 110 mg/dL at 8-10 weeks, it may help identify pregnant women who are at risk of developing GDM [4,5]. Prediction of GDM by two-hour PPBG >110 mg/dL will facilitate early appropriate intervention to prevent the development of GDM and hyperinsulinemia [5]. The rationale behind early prevention lies in the recognition that GDM is not merely a transient condition confined to the gestational period; rather, it serves as a forerunner of risks for the development of type 2 diabetes mellitus in the mother and predisposes offspring to metabolic and cardiovascular complications later in life.

This pilot study was planned to examine the above concept and determine if, at 8-10 weeks of gestation, two-hour PPBG or HbA1c could predict GDM. Pregnancy outcomes were also evaluated in these women.

## Materials and methods

This prospective observational cohort study was done at Lady Hardinge Medical College and associated hospitals in New Delhi, India, after approval from the Institutional Ethics Committee (IEC) (LHMC/IEC/2022/PG thesis/18 dated 27/09/2022). The study was conducted in accordance with the International Council for Harmonisation (ICH) - Good Clinical Practice (GCP) (ICH-GCP) guidelines. Consecutive pregnant women with a gestation period between 8 and 10 weeks were enrolled in the study from antenatal OPD after written informed consent. Women with pre-existing diabetes mellitus or polycystic ovary syndrome (PCOS) on metformin therapy or having bleeding or pain in the abdomen were excluded.

A detailed history and examination were performed. All recruited pregnant women underwent testing for two-hour PPBG and HbA1c at 8-10 weeks. GDM screening was performed at 14-16 weeks, 24-28 weeks, and 32-34 weeks. GDM was diagnosed by the Diabetes in Pregnancy Study Group of India (DIPSI) test if blood glucose is > 140 mg/dL after two hours of 75 gm glucose challenge, irrespective of fasting status. If at any time a woman was detected to be having GDM, standard treatment for gestational diabetes was initiated without subjecting her to additional GDM testing. All women were followed up until delivery to determine the association of two-hour PPPBG and HbA1c with fetomaternal outcome. Cord blood was collected to measure glucose and insulin levels in the neonate.

The sample size was calculated based on an earlier study conducted by Poo et al. [3]. The sensitivity of first-trimester abnormal glucose in predicting GDM was 82.4%, and the prevalence of GDM in their study was 11.3%. Based on these parameters, the sample size is estimated as 123 by using the formula: TP + FN = zα2 (SN(1-SN)) / d2. Considering that many patients would be lost to follow-up or may undergo abortion, 200 subjects were recruited.

Data were analyzed using Statistical Product and Service Solutions (SPSS, version 21.0; IBM SPSS Statistics for Windows, Armonk, NY). Data were computed as mean and SD standard deviation, median, and interquartile range if the data were continuous. Data were presented as percentages if they were categorical. An unpaired t-test was done to compare the two groups' mean or median. The chi-square or Fisher's exact test was done to determine the association between nonmodified variables. Pearson correlation was used to find a correlation between two continuous variables. The screening test was validated by using receiver operating characteristic (ROC) curve analysis to calculate diagnostic accuracy, sensitivity, specificity, PPV, and NPV; p-value of less than 0.05 was considered significant.

## Results

Out of 200 pregnant women, five had a spontaneous abortion before 10 weeks, and six patients were lost to follow-up; 189 patients were followed up till delivery with a mean gestational age of 9.03+0.57 weeks at enrollment.

The mean age of women with PPBG < 110 mg/dL was significantly lower than that of women having PPBG > 110 mg/dL (25+4.1 vs 27.5+4.3 years; p < 0.001). Women having PPBS > 110 mg/dL had a significantly higher mean BMI as compared to women having PPBG < 110 mg/dL (23±1.6 versus 22.2+1.09 kg/m^2^; p < 0.001). The proportion of women with a past history of abortions, family history of diabetes, and PCOS in both groups is compared in Table 1.

**Table 1:** High-risk factors

Past history	<110 mg/dL (n=92)	>110 mg/dL (n=97)	Total (n= 189)	P value
History of abortion	7 (7.61%)	31 (31.96%)	38 (20.11%)	0.0001
History of PCOS	4 (4.35%)	15 (15.46%)	19 (10.05%)	0.014
Family history of DM	4 (4.35%)	28 (28.87%)	32 (16.93%)	0.0001
History of still birth	0 (0%)	3 (3.09%)	3 (1.59%)	0.247

In women having PPBS < 110 mg/dL at 8-10 weeks, two (2.17%) women developed GDM at 12-14 weeks, one (1.08%) each at 24-28 weeks, and at 32-34 weeks. In women having PPBS > 110 mg/dL at 8-10 weeks, 71 (73.19%) women developed GDM at 12-14 weeks, 15 (15.46%) at 24-28 weeks, and seven (7.216%) developed GDM at 32-34 weeks.

Ninety-seven (51.32%) women developed GDM. Of these, 93 (95.88%) had PPBG > 110 mg/dL as compared to four (4.35%) women with PPBG < 110. Out of 97 GDM patients, 73 were well-controlled on diet, 13 required metformin, and 11 were controlled on insulin.

A significantly higher proportion of women in PPBG > 110 mg/dL developed hypertensive disorders of pregnancy (p < 0.001) and dyslipidemia (0.014). The distribution of medical disorders is depicted in Table 2.

**Table 2:** Comparison of medical disorders

High-risk factor	<110 mg/dL (n=92)	>110 mg/dL (n=97)	Total (n= 189)	P value
Hypertensive disorders of pregnancy	8 (8.61%)	27 (27.68%)	29 (15.34%)	0.004
IHCP	19 (20.65%)	26 (26.80%)	45 (23.81%)	0.321
Dyslipidemia	2 (2.17%)	22 (22.68%)	24 (12.7%)	0.001

The number of women with polyhydramnios (p = 0.247), preterm deliveries (p = 0.373), and the rate of cesarean section (p = 0.023) was higher in women with the PPBG > 110 mg/dL group. A significantly higher number of women with PPBG > 110 mg/dL had newborns with birth weight less than 2.5 kg (p < 0.001), as well as with birth weight > 3.5 kg (p < 0.001) (Table 3).

**Table 3:** Comparison of obstetric and neonatal outcomes

Baby birth weight (kg)	<110 mg/dL (n=92)	>110 mg/dL (n=97)	Total (n= 189)	P value
Polyhydramnios	0 (0%)	3 (3.09%)	3 (1.59%)	0.247
Preterm labour	2 (2.17%)	22 (22.7 %)	24 (12.70%)	0.0001
Vaginal delivery	75 (81.52%)	65 (67.01%)	140 (74.07%)	0.023
LSCS	17 (18.48%)	32 (32.99%)	49 (25.93%)	
Birth weight <2.5 kg	16 (17.39%)	38 (39.18%)	54 (28.57%)	0.0009
Birth weight >3.45 kg	9 (9.78%)	30 (30.93%)	39 (20.63%)	0.0003

**Figure 1:**
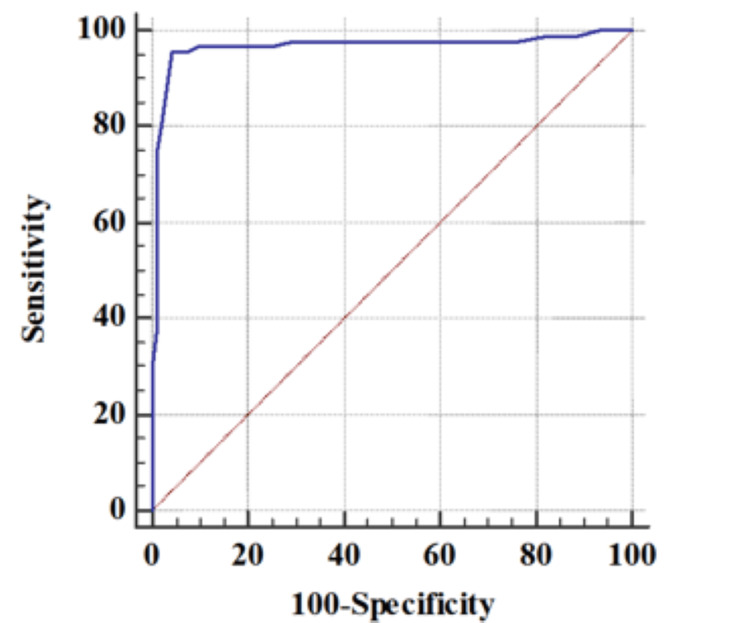
Receiver operating characteristic (ROC) for predicting gestational diabetes mellitus (GDM) by two-hour postprandial blood glucose (PPBG)

Predictive performance: The area within the ROC curve (AUC) for PPBG was 0.969, indicating excellent discriminatory power for identifying GDM. Similarly, the AUC for HbA1c was 0.916, demonstrating positive predictive performance (Figure 1).

Optimal cutoffs: The optimal cutoff values for predicting GDM were > 110 mg/dL for PPBG and > 5.3% for HbA1c. These cutoff values represent thresholds above which individuals are at increased risk of GDM. PPBG has been tested for the first time as a predictor of the development of GDM based on DIPSI guidelines [3]. HbA1c >5.3% has also been suggested as a predictor of GDM in a previous study [1].

Diagnostic performance: PPBG exhibited high sensitivity (95.88%) and specificity (95.65%), indicating its ability to identify both true positive and negative cases of GDM accurately. HbA1c also demonstrated good sensitivity (81.44%) and specificity (85.87%), although lower than PPBS.

Diagnostic accuracy: PPBG achieved a diagnostic accuracy of 95.77%, while HbA1c exhibited a slightly lower accuracy of 83.60%, highlighting the superior overall performance of PPBG in predicting GDM compared to HbA1c (Table 4).

**Table 4:** Diagnostic accuracy of postprandial blood glucose (PPBS) and HbA1c for predicting gestational diabetes mellitus (GDM) POG: Period of gestation

Gestational diabetes mellitus	PPBS (mg/dL) (2 hours) at <12 weeks of POG	HbA1c (%) at <12 weeks of POG
Area under the ROC curve (AUC)	0.969	0.916
Standard error	0.0145	0.0193
95% Confidence interval	0.933-0.988	0.867-0.951
P value	<0.0001	<0.0001
Cutoff	>110 mg/dL	>5.3%
Sensitivity (95% CI)	95.88% (89.8-98.9%)	81.44% (72.3-88.6%)
Specificity (95% CI)	95.65% (89.2-98.8%)	85.87% (77.0-92.3%)
PPV (95% CI)	95.9% (89.8-98.9%)	85.9% (77.0-92.3%)
NPV (95% CI)	95.7% (89.2-98.8%)	81.4% (72.3-88.6%)
Diagnostic accuracy	95.77%	83.60%

## Discussion

In the present study, more than half of the participants had GDM. A significantly higher proportion of women in the younger age group had PPBS < 110 mg/dL than the older group (p < 0.049). Previous studies have also found that advancing maternal age was independently correlated with a high risk of GDM, possibly due to the age-related progressive nature of beta cell dysfunction and increased insulin resistance. Age > 25 years had a significant association with GDM [6-8]. Women having PPBS > 110 had a significantly higher BMI as compared to women with PPBS < 110.

Women with PPBG > 110 mg/dL had a significantly higher proportion of women with a history of abortions, family history of diabetes, and PCOS, as also observed in previous studies [6-9].

In the present study, a significantly higher proportion of women with PPBG > 110mg/dL had preterm delivery compared to women with PPBG < 110 mg/dL. Cesarean deliveries due to fetal-pelvic disproportion, macrosomia, prolonged labor, and second-stage arrest were higher in women with PPBG > 110 mg/dL. Dhutraj et al. [10] in their study observed that 28.41% had lower segment cesarean section due to macrosomia, cephalopelvic disproportion, obstructed labor, and fetal distress.

In the present study, 20.63% of newborns had birth weight > 3.5 kg. Women who had PPBG > 110 mg/dL had a higher proportion of newborns with birth weight > 3.5 kg compared to those with PPBG < 110 mg. Overall, 28.57% of newborns had a birth weight of less than 2.5 kg. Women with higher PPBS levels > 110 mg/dL had a higher proportion of newborns with birth weight < 2.5 kg compared to those with lower PPBS levels. Of these, 22.7% were low birth weight because they were born prematurely. Other reasons for low birth weight in this group were associated with hypertensive disorders of pregnancy. Previous studies [8,10] also found a higher magnitude of hypertension, macrosomia, cephalopelvic disproportion, and cesarean delivery in women with hyperglycemia. 

A positive correlation was observed between two hours of PPBG and cord blood glucose levels. A positive correlation was also present between insulin levels measured during early pregnancy and cord blood insulin levels at birth. This indicates that maternal hyperglycemia in the initial weeks determines the glycaemic status of the offspring by crossing the placental barrier leading to metabolic programming.

Amongst women with PPBS > 110 mg/dL, 95.88% of them developed GDM, while in a group with PPBS < 110 mg/dL, only 4.12% developed GDM. This association was highly significant, indicating that PPBG > 110 mg/dL strongly predicted GDM.

Even though glucose is essential for the growth of a developing fetus, it harms the growing fetus. It causes it to become hyperglycemic, giving rise to a hypothesis called "fuel-mediated teratogenesis," and proposing the explanation for the association of excessive fetus growth. Maternal insulin does not cross the placenta freely, while maternal glucose does, and in response, the fetal beta cell mass of the pancreas tries to balance by secreting more insulin. Insulin acts as a fetal growth hormone and becomes responsible for promoting growth and adiposity. Intrauterine exposure to hyperglycemia has harmful effects in addition to those related to genetic predisposition [11].

The DIPSI guidelines [4] have recently subcategorized different degrees of glucose intolerance during pregnancy. Early gestational glucose intolerance (EGGI) is defined as PPBS > 110 mg/dL before the ninth week of gestation and has indicated that these women are prone to develop GDM later during the course of pregnancy. The current study has proven this novel concept.

The pathophysiological relationship between maternal glucose and fetal neonatal adiposity is mediated through fetal insulin, and there is a direct relationship between maternal glucose measured as early as 8-10 weeks gestation and the risk of a macrosomic newborn. The human pancreas begins to develop four weeks after conception and first insulin deposits can be found between seven and eight weeks [11]. The fetal renal threshold for glucose is 110 mg/dL. When glucose levels exceed this value, it results in glycosuria and increases insulin levels in the amniotic fluid. Amniotic fluid insulin is detectable at 12 weeks and can be used as a surrogate for fetal serum insulin [11,12]. Amniotic fluid insulin is highly correlated with cord blood insulin measured at birth. It is to be remembered that fat deposition in the fetus begins at approximately 14 weeks, which is the duration of onset of early fetal hyperinsulinemia [11-14]. Adverse neonatal outcomes are high for mothers with hyperglycemia during preconception and early gestation periods, often despite seemingly optimum glycaemic control in the latter half of pregnancy, which could be a consequence of the early establishment of fetal hyperinsulinemia, stimulus exaggerates the fetal glucose steal [11].

Essentially, fetal hyperinsulinemia lowers fetal glucose levels and increases the glucose concentration across the placenta and, subsequently, the glucose flux to the fetus [11]. While the slope of this gradient and glucose flux will be greatest when maternal hyperglycemia and fetal hyperinsulinemia coexist, fetal hyperinsulinemia will favor a persistently high glucose flux even when maternal blood glucose is normal [11-13]. The obvious interpretation is that glycaemic control needs to be regulated very early in pregnancy to prevent the establishment of fetal hyperinsulinemia, again supporting the need for pre-pregnancy planning and timely establishment of maternal glycaemic control. An exaggerated glucose steal by a hyperinsulinemic fetus could also lower maternal glucose levels during an oral glucose tolerance test (OGTT), explaining why some mothers with fetuses exhibiting all the features of diabetic fetopathy have 'normal' glucose tolerance [14].

In the present study, the diagnostic accuracy of two-hour PPBS was compared with HbA1c for the prediction of GDM. The high diagnostic accuracy of PPBG > 110 mg/dL suggests its potential utility as a screening tool for identifying individuals at risk of GDM during early pregnancy. This simple physiological test can even be done at home or primary healthcare centers using a glucometer, as 70% of India's vast population resides in villages and does not have access to reliable lab facilities. Early identification of individuals at risk of GDM based on PPBG can facilitate timely intervention and management strategies, thereby reducing the risk of adverse maternal and fetal outcomes associated with untreated GDM.

The study's limitations include the need for a multicentric study to ensure the generalization of the findings. A longer follow-up cohort of these mothers and their newborns would be useful in evaluating the long-term implications.

Preventive measures against NCD should start during the preconception or intrauterine period and continue throughout life from early childhood [15,16]. Similarly, prevention of type 2 diabetes mellitus must begin in the uterus and continue throughout the life course.

## Conclusions

PPBG > 110 mg/dL at two hours exhibits a diagnostic value, sensitivity, specificity, positive predictive value (PPV), and negative predictive value (NPV) of > 95% for the prediction of GDM. PPBG was observed to be a superior, physiologic, and low-cost option than HbA1c for early prediction of GDM and correlated with adverse medical and obstetrical outcomes. It can also be done as a simple point-of-care test with a glucometer at home or in the periphery by healthcare workers (HWCs) and in wellness centers.

Therefore, early identification of individuals at risk of GDM based on PPBG > 110 mg/dL may facilitate timely intervention and management strategies, thereby reducing the further risk of adverse maternal and fetal outcomes.
